# The Influence of Volume Conduction on DTF Estimate and the Problem of Its Mitigation

**DOI:** 10.3389/fncom.2017.00036

**Published:** 2017-05-12

**Authors:** Maciej Kaminski, Katarzyna J. Blinowska

**Affiliations:** ^1^Department of Biomedical Physics, Faculty of Physics, University of WarsawWarsaw, Poland; ^2^Institute of Biocybernetics and Biomedical Engineering of Polish Academy of SciencesWarsaw, Poland

**Keywords:** volume conduction, connectivity measures, directed transfer function, source space analysis, sensor space analysis

The multivariate frequency dependent methods based on Multivariate Autoregressive Model (MVAR) and Granger causality principle: Directed Transfer Function (DTF) (Kaminski and Blinowska, 1991), its modification full frequency DTF (ffDTF) (Korzeniewska et al., 2003), and Partial Directed Coherence (PDC) (Baccala and Sameshima, 2001) have been widely used for estimation of brain activity transmission patterns, since they supply information about causal relations between signals. DTF_*ij*_(*f*) describes propagation in frequency *f* from channel *j* to channel *i* normalized in respect to propagations from all other channels. For ffDTF the normalization factor is integrated over full frequency band, which makes the normalization frequency independent. PDC_*ij*_(*f*) is based on transformation to frequency domain MVAR model coefficients (unlike DTF, which is based on the transfer function of the MVAR). PDC shows direct flows from channel *j* to *i* and it is normalized in respect to outflows from channel *i*.

Recent papers (Brunner et al., 2016; Van de Steen et al., 2016) appeared pointing out that DTF based on scalp measurement is not free of volume conduction effect as claimed in Kaminski and Blinowska (2014). We have to admit that indeed a mixing of cortical sources activities influences results of DTF calculated in a sensor space, however this effect does not undermine critically DTF results. The dipole electromagnetic field decays fast with the distance which mitigates the volume conduction effect.

In the example given in Van de Steen et al. (2016) (Simulation I), where two independent sources were considered, the off diagonal elements of mixing matrix were assigned arbitrarily to very high values, in consequence yielding strong interdependences of sources as estimated by DTF. In practice, due to the decrease of the dipole field with a distance, this mixing doesn't have such a high value.

Simulation II in Van de Steen et al. (2016) is based on a rather unrealistic assumption that one of two sources drives another source with a specific delay. The activity generated in concrete brain structures is a result of an action of distinct neural populations, which work on their own pace. They may exchange the information by a transmission of electrical activity, but it is very unlikely that one source drives another constantly with a specific delay.

The simulation presented in Brunner et al. (2016) is a more realistic one. In the simulated example the strengths of the connections caused by the mixing are almost an order of magnitude smaller than the true connections. We admit that spurious connections may be generated due to the mixing caused by the volume conduction, but their strengths are usually much smaller than the true connections as shown in the simulated example. Indeed, the volume conduction effects are present, however their contribution is of a secondary importance in comparison to the DTF-values indicating effective transmission.

It is worth reflection that in practice results of DTF analysis correspond very well with known neuroimaging, intracranial and electrophysiological evidence including topographic accuracy (Ginter et al., 2001; Kuś et al., 2006; Blinowska et al., 2010, 2013; Brzezicka et al., 2010; Wyczesany et al., 2014, 2015; Ligeza et al., 2016). As an example may serve the finger movement task. The topographical and time-frequency characteristics of this task were extensively studied and described in terms of so-called desynchronization and synchronization phenomena (ERD/ERS) (Pfurtscheller and Lopes da Silva, 1999). One may expect that the propagation of EEG activity should correspond to this well established evidence.

Indeed in our studies based on scalp EEG we have observed as expected: decrease of propagation in the alpha and beta band during movement (ERD) from the electrodes overlying primary motor cortex (PMC) followed by an increase in propagation (ERS) from these sites. Also a burst of propagation in gamma band initializing the movement was found exactly from electrode overlying PMC contralateral to the moving finger. Interestingly, during an imagination of the movement a cross-talk between primary and supplementary motor areas was observed (animations showing topographical time-frequency dependent propagation are available at: https://brain.fuw.edu.pl/~kjbli). Moreover, the so-called surround effect—increase of activity from electrodes placed around, but not over PMC of moving finger—was also mirrored by the propagations from the relevant sites (Suffczynski et al., 1999; Ginter et al., 2001), which testifies for a very good topographical resolution of the DTF estimate. The topographical accuracy of all these observations was determined by the spacing of electrodes (10–10) system, within these bonds there was a perfect correspondence with the known phenomena described in Pfurtscheller and Lopes da Silva (1999).

The finger movement was also studied in Babiloni et al. (2005), where a projection to the cortex was performed before application of DTF. The EEG from 96 electrodes was recorded, but the resolution of the obtained topographical information was limited to several regions of interest (ROIs). In Babiloni et al. (2005) only minimal changes of connectivity were observed between pre- and post-movement periods. The observed flows originated in the very large cortex areas. The gamma outflow from the hemisphere ipsilateral to the moving finger was reported, contrary to the known evidence that it is the contralateral hemisphere which is involved in the movement initialization. Summarizing, there was no correspondence of the results with the established evidence (Pfurtscheller and Lopes da Silva, 1999).

In the paper concerning foot movement (De Vico Fallani et al., 2008), where the projection of sensor signal to cortex was performed, also no correspondence of propagation estimated by Partial Directed Coherence (PDC) with a known ERD/ERS phenomena was observed. Surprisingly, no change of network configuration was found in the alpha band, which is known as the most reactive rhythm in the movement execution.

In order to understand these discrepancies we have compared results of DTF applied to the EEG sensor time series and to their projection on the cortex, during the finger movement task (the details of the recording may be found in Ginter et al., 2001). We have applied the standardized Low Resolution Electromagnetic Tomography (sLORETA) (Pascual-Marqui, 2002) to the sensor time series to obtain cortical EEG activity. In order to find regions of cortex corresponding to given electrodes we have used projections of electrode positions onto the cortical surface according to Koessler et al. (2009), where cranio–cerebral correlations for the 10–10 system were studied. For right finger movement we have calculated ffDTF functions for left hemisphere for sensor signals and source signals obtained by sLORETA. We compared epochs 0–1 and 1–2 s, where 0 is a time of movement onset. In Figure 1 the ffDTF functions and the corresponding EEG propagations illustrated by arrows are shown for both approaches. Upper part of the figure shows in each small box ffDTF functions in 0–20 Hz frequency range. They describe transmission from the electrode marked below the column to the electrode marked at the left of the given row.

**Figure 1:**
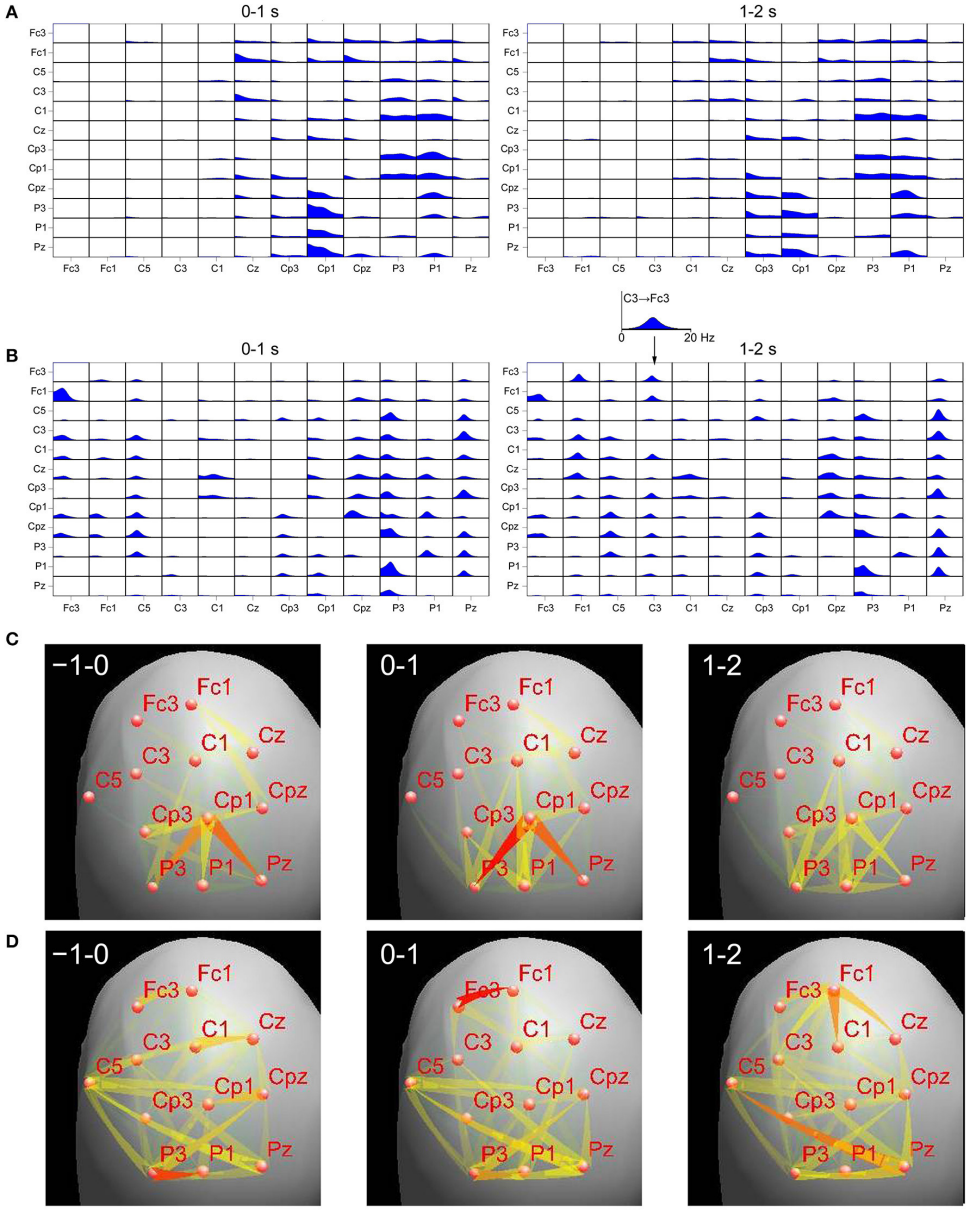
**Comparison of transmissions during right finger movement obtained by means of ffDTF from signals projected to the cortex (A,C)** and scalp signals **(B,D)**, for two epochs. Top panels: **(A)**—ffDTF function calculated for signals projected to the cortex, **(B)**—ffDTF calculated for sensor signals. In each small box ffDTF as a function of frequency (from 0 to 20 Hz) is shown. The flows are from the electrode marked below the picture to the electrode marked at the left. Insert shows enlarged example of ffDTF for transmission from C3 to Fc3. At the left the results during the movement (0–1 s, where 0 is the movement onset); at the right propagation after the movement (1–2 s). Bottom panels: flows of EEG activity (ffDTF functions integrated in the frequency range 5–20 Hz). **(C)**—Signals projected to the cortex and **(D)**—Sensor signals. The strength of the flow indicated by the intensity and color of the arrow (red the strongest). In the upper right corners of the panels time in seconds in respect to movement offset (second 0) are marked. Narrow end shows destination electrode.

In case of cortex projected signals (Figures 1A,C) we can hardly see any differences depending on the epoch. The propagation from Cp3 is hard to interpret in terms of the known evidence. Especially surprising is the lack of propagation from C3 in the epoch 1–2 s corresponding to the known resynchronization effect (Figure 1A, right panel).

In case of sensor signals (Figures 1B,D) in the epochs corresponding to the movement preparation and execution the so-called surround effect—propagation from electrodes surrounding C3 (electrode overlying motor cortex of the right finger), but not from C3 (desynchronization) is visible. In the next epoch the resynchronization effect can be perceived as an increased propagation from centro-frontal structures and also from C3. More accurate illustration of the evolution of propagation in time-frequency space obtained from sensor signals during movement may be found in Ginter et al. (2001) and is also illustrated by animations (http://brain.fuw.edu.pl/~kjbli).

The above results are just an example and cannot be considered as a proof of a lack of influence of volume conduction on DTF, but they indicate that this influence is weak and doesn't have significant effect on the results. The question arises: is the application of pre-processing procedures, which mix information from the channels of the system, a right solution? Do the procedures projecting the scalp electrical activity to the cortex reproduce well the phase structure of the cortical sources? Such question seems to be supported by weak correspondence with the known evidence of the propagation results obtained from reconstructed signals. The results of Simulation II in reference (Van de Steen et al., 2016) do not prove the correctness of approach based on projection of signals to scalp, since in simulation the same head model was used.

Inferring from scalp electrodes the underlying cortical activity is a hard problem complicated by the non-uniqueness of its solutions. In fact the head models are only approximations of “true” head structures. The reconstruction procedures are based on a prior knowledge of the sources. The inverse solutions depend on informed constraints and the accuracy of results is limited by the accuracy of these constraints. The Authors of Van de Steen et al. (2016) admit themselves that the inverse solution does not undo the mixing completely and hence spatial leakage will still be present. Different methods have been proposed to solve the inverse problem applying idealized or realistic head models, assuming dipoles or distributed sources. These methods allowed to estimate with bigger or smaller accuracy the strength and position of the cortical sources from signals measured at scalp, reducing to the certain extent the effects of volume conduction, however the reconstruction of the true phase patterns of real cortical sources may not be such a straightforward problem. Taking into account above considerations the advantages of the approach based on reconstructed source space signals for estimation of connectivity are not very convincing.

Spatial propagation of volume conduction was studied in Nunez and Srinivasan (2006). They estimated theoretically and experimentally the decay with the distance of coherences due solely to volume conduction. The results showed that at the distance of 7 cm (roughly the distance between electrodes in 10–20 system) these coherences were close to zero. Therefore, we may assume that mixing of cortical sources activities due to volume conduction doesn't influence the DTF results in a significant way.

Again, we admit that DTF calculated from scalp electrodes can be influenced by volume conduction, however in practice this influence does not distort substantially the estimates and by applying a proper threshold on the obtained propagation values one can get correct results. As an argument for this kind of approach may serve very good agreement of the DTF results with the known evidence supported by numerous publications, some of them mentioned already above.

It would be interesting to undertake the study, which would clarify the problem of phase reconstruction in the source space from scalp signals, because neither our examples nor examples with recalculated sources/simulations can be considered a definite proof. Probably it would require simultaneous measurements of intracranial and scalp signals. We think that the problem of estimation of effective connectivity and choice of the proper approach to pre-processing is open and requires further studies.

## Author contributions

The authors MK and KB contributed to the design of the work and interpretation and discussion, revised the manuscript, approved the final version, and agreed to be accountable for all aspects of the work.

## Funding

This work was supported by the Statutory Grant of Polish Ministry of Science and Higher Education to Faculty of Physics of University of Warsaw (MK) and Statutory Grant of Polish Ministry of Science and Higher Education to Institute of Biocybernetics and Biomedical Engineering (KB).

### Conflict of interest statement

The authors declare that the research was conducted in the absence of any commercial or financial relationships that could be construed as a potential conflict of interest.
